# Novel Eye Movement Disorders in Whipple’s Disease—Staircase Horizontal Saccades, Gaze-Evoked Nystagmus, and Esotropia

**DOI:** 10.3389/fneur.2017.00321

**Published:** 2017-07-11

**Authors:** Aasef G. Shaikh, Fatema F. Ghasia

**Affiliations:** ^1^Daroff-Dell’Osso Ocular Motility Laboratory, Cleveland VA Medical Center, Cleveland, VA, United States; ^2^Department of Neurology, Case Western Reserve University, Cleveland, OH, United States; ^3^Neurological Institute, University Hospitals, Cleveland, OH, United States; ^4^Neurology Service, Louis Stokes, Cleveland, VA, United States; ^5^Cole Eye Institute, Cleveland Clinic, Cleveland, OH, United States

**Keywords:** neurodegeneration, progressive supranuclear palsy, slow saccade, parkinsonism, strabismus

## Abstract

Whipple’s disease, a rare systemic infectious disorder, is complicated by the involvement of the central nervous system in about 5% of cases. Oscillations of the eyes and the jaw, called oculo-masticatory myorhythmia, are pathognomonic of the central nervous system involvement but are often absent. Typical manifestations of the central nervous system Whipple’s disease are cognitive impairment, parkinsonism mimicking progressive supranuclear palsy with vertical saccade slowing, and up-gaze range limitation. We describe a unique patient with the central nervous system Whipple’s disease who had typical features, including parkinsonism, cognitive impairment, and up-gaze limitation; but also had diplopia, esotropia with mild horizontal (abduction more than adduction) limitation, and vertigo. The patient also had gaze-evoked nystagmus and staircase horizontal saccades. Latter were thought to be due to mal-programmed small saccades followed by a series of corrective saccades. The saccades were disconjugate due to the concurrent strabismus. Also, we noted disconjugacy in the slow phase of gaze-evoked nystagmus. The disconjugacy of the slow phase of gaze-evoked nystagmus was larger during monocular viewing condition. We propose that interaction of the strabismic drifts of the covered eyes and the nystagmus drift, putatively at the final common pathway might lead to such disconjugacy.

## Introduction

Whipple’s disease is a rare systemic disorder caused by a Gram-positive bacterium *Tropheryma whippelli* (1, 2). Although malabsorption syndrome is the typical manifestation of the Whipple’s disease, in 5% of cases it primarily and initially involves the central nervous system (3). Prominent cognitive symptoms including hypersomnolence had led to the identification of central nervous system Whipple as a form of “unclassifiable encephalitis” (4). In addition to robust cognitive dysfunction and hypersomnolence; complete vertical ophthalmoplegia, approximately 1 Hz convergent–divergent eye oscillations, and concurrent contractions of the masticatory muscles are the hallmark of the central nervous system Whipple’s disease (5–7). The Whipple’s disease can present without its classic manifestations, but with prominent parkinsonism and slowing and curved trajectories of vertical saccades (8, 9). The selective deficit of the vertical saccades in patients with Whipple’s disease were attributed to the involvement of the rostral interstitial nucleus of the medial longitudinal fasciculus (riMLF), the anatomical substrate for vertical saccade generation (10). While vertical saccades in Whipple’s disease are prominently affected, the horizontal saccades generated at paramedian pontine reticular formation are also often affected (8, 9).

Here, we present the quantitative study of abnormal horizontal saccades in Whipple’s disease. In addition to quantitatively investigating the horizontal saccades in Whipple’s disease, our study delves into the pathophysiology of series of hypometric saccades (staircase saccades) in Whipple’s disease. Delineating these mechanisms will facilitate our understanding of the pathophysiology and heterogeneity of saccadic disorders. The objective measures will provide reliable and possible prodromal disease markers that will help the early differential diagnosis of disorders affecting the saccade velocity. Our Whipple’s disease patient also had two other atypical features—gaze-evoked eye nystagmus and esotropia.

## Methods

### Clinical Description

A 73-year-old man with a history of Whipple’s disease presented for diplopia, change in gait, imbalance, memory loss, behavioral changes, and diarrhea. Cognitive examination revealed the score of 21/30 on Montreal Cognitive Assessment (MoCA, version 7.3). Examination of cranial nerves revealed vertical gaze restriction, absent vertical optokinetic nystagmus, however, vestibulo-ocular reflex was intact vertically and horizontally. The saccade latency was 276 ± 23 ms; increased compared to normal 185 ± 16 ms (*t*-test, *p* < 0.01). His horizontal saccades had multiple interruptions (staircase saccade). The saccade gain (achieved gaze-shift amplitude/desired gaze-shift amplitude) was 0.45 ± 0.16; which was significantly lower compared to normal 0.91 ± 0.02 (*t*-test, *p* < 0.01). He had 10 prism diopters of esotropia with no distance-near disparity and greater limitation of abduction than adduction. The esotropia contributed to post-saccadic drifts, which were more pronounced under monocular viewing conditions. He also had gaze-evoked nystagmus. We did not notice skew or alternating skew deviation or vertical nystagmus. The vestibulo-ocular reflex cancelation was technically difficult to perform due to increased neck tone secondary to coexisting parkinsonism. We did not get an ideal assessment of pursuit system due to overlying gaze-holding deficits. Nevertheless, our clinical impression was that the pursuit system had lower gain. He had face dystonia, but there were no rhythmic contractions of the face or the jaw. He had mild retrocollis. There was an increase in axial and appendicular tone, bradykinesia, and hypokinesia. There was pronounced shuffling of gait and reduction in arm swings on both sides. He scored 22 points on Unified Parkinson’s Disease Rating Scale (axial score 10; left side 6 and right side 5). The diagnosis of Whipple’s disease was established with colonoscopy and biopsy performed for the investigation of chronic diarrhea. The study revealed blunting of intestinal villi and lamina propria. There were large numbers of PAS-positive, diastase-resistant foamy macrophages. The patient had a cardiac pacemaker. Hence, brain MRI was not performed. He was then treated with intravenous ceftriaxone and has been on sulfamethoxazole.

### Eye Movement Measurements

The experiment protocol adhered declaration of Helsinki, and it was approved by the Cleveland Clinic institutional review board. The subject and his legal guardian gave written informed consent for the experiment and publication of results. Binocular horizontal and vertical eye positions were captured non-invasively at 500 Hz sampling rate using video-based eye tracker (EyeLink 1000^®^, SR Research, ON, Canada). However, given limitation to vertical gaze, we only analyzed horizontal saccades. In monocular viewing condition, one eye was covered with the infrared permissive filter. This filter allowed infrared waves but blocked visible light waves, hence, preventing vision through the covered eye. The infrared permissive filter allowed measurement of the position of the covered eye. Technique and experimental protocol used to measure the positions of both eyes were otherwise identical for binocular and monocular viewing conditions. The details of data acquisition, signal processing, and analysis were similar as outlined in our previous studies (11–15).

## Results

**Figure 1:**
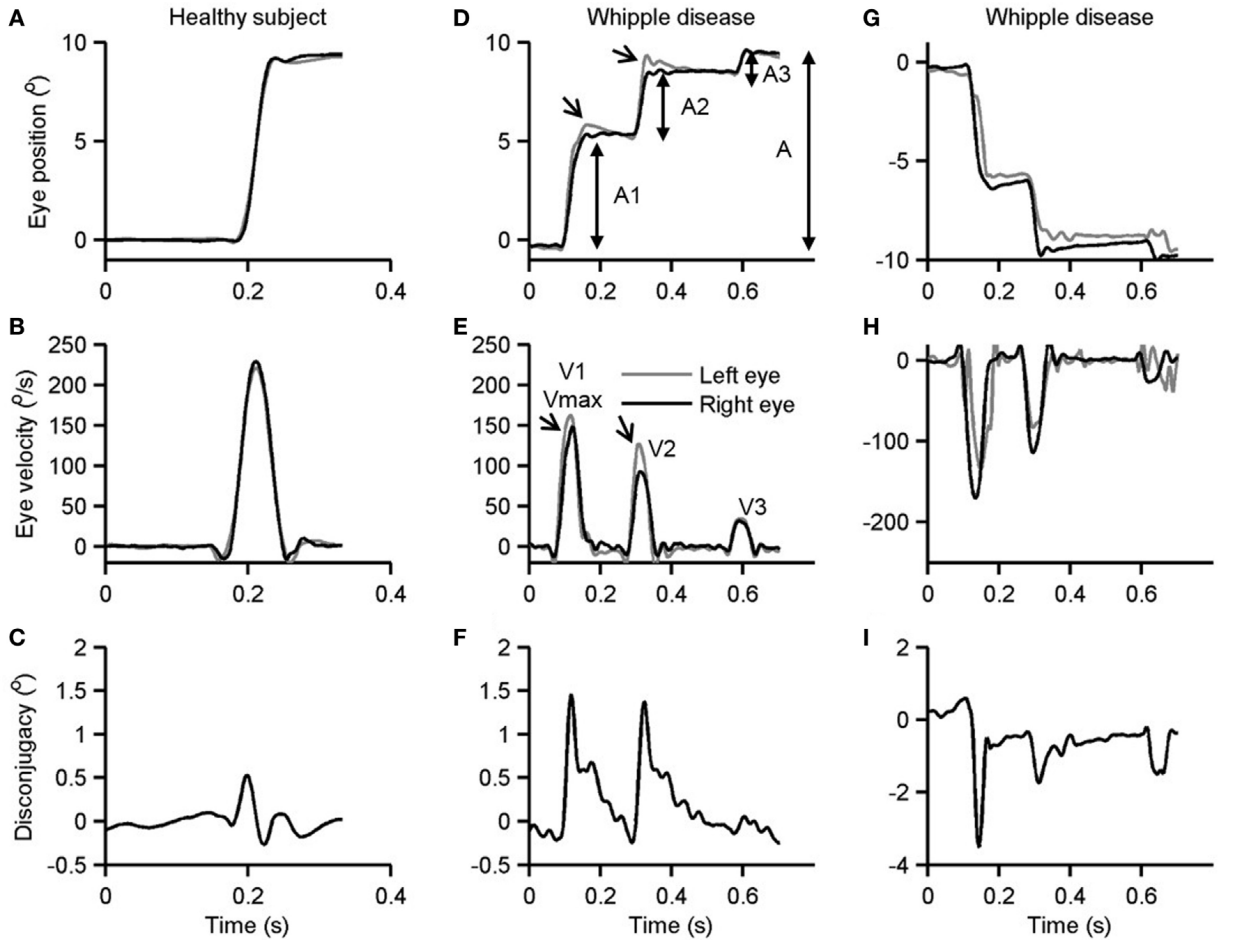
Example of single visually guided saccade from a healthy subject **(A–C)** and its comparison in a patient with Whipple’s disease. Panels **(A,D,G)** depict horizontal eye position plotted along *y*-axis while corresponding time on the *x*-axis. Panels **(B,E,H)** depict horizontal eye velocity plotted on the *y*-axis and corresponding time on the *x*-axis. Panels **(C,F,I)** illustrate the difference in right and left horizontal eye position. Arrows in panels **(D,E)** depict disconjugacy in a segmented saccade.

Figure 1 illustrates an example of a visually guided saccade shifting the gaze from straight-ahead to 10° to the right and to the left in the patient with Whipple’s disease and its comparison with a healthy subject. Figure 1A is an example of a healthy subject. In this example, the subject shifted gaze to 10° to the right side. Binocular eye positions measured during this example in healthy subject depicts uninterrupted gaze shift in 60 ms timespan (Figure 1A). As depicted in Figure 1C, the eye velocity during such gaze shift had a single peak. The right and the left eyes moved at a comparable amplitude (Figure 1A) and velocity (Figure 1C); there was no disconjugacy. Figures 1D,G show similar gaze shift in the patient with Whipple’s disease during rightward and leftward saccades, respectively. The gaze shift in the patient took 530 ms for the right side and 535 ms for the left. The saccades were frequently interrupted as shown in an example in Figure 1D. During each break, the eye velocity reached 0 (Figures 1E,H). Such complete pause in eye movement is seen in Figures 1D,G and further emphasized as individual peaks and complete pause in Figures 1E,H. Also, the eye positions and velocities were not conjugate during such shift. The left eye had larger shift during each segment of the rightward saccade and *vice versa* for the leftward saccade. Such shifts are followed by a post-saccadic drift thereby fusing the gaze from both eyes. Figures 1F,I emphasize disconjugacy in the saccade amplitude.

### Amplitude Velocity Relationship

**Figure 2:**
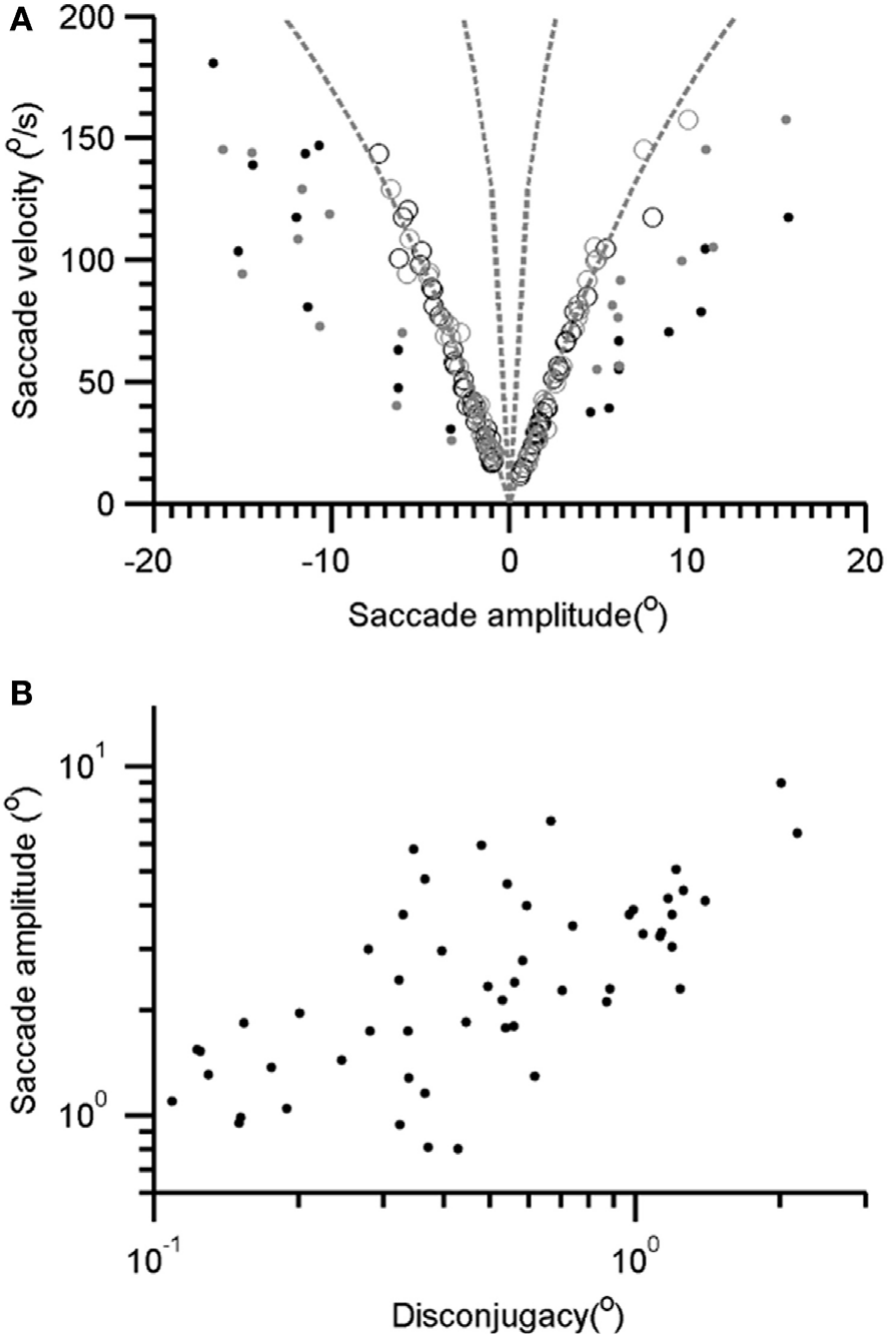
**(A)** Amplitude to velocity relationship of the horizontal saccade. The open symbols depict such relationship for segmented saccade (V1 and A1 relationship, see Figure 1C). Filled symbols depicted the relationship of maximal velocity and desired gaze shift (Vmax and A relationship, see Figure 1C). Gray symbols depict rightward while black symbols are leftward saccades. **(B)** Comparison of segmented saccade amplitude and corresponding disconjugacy. Each data-point depicts one segmented saccade.

As depicted in Figures 1D–I, the saccades in our patient are frequently interrupted, or they fall short of the target. Subsequently, there are catchup saccades that shift the eyes to reach the desired position. To quantitatively examine this phenomenology, we measured the kinematics of the visually guided horizontal saccades in patients with Whipple’s disease and compared them with normal. The interruption in the visually guided saccades can be explained by three possible mechanisms. According to one mechanism, the saccade command is mal-programmed, and the executed gaze shift (saccade amplitude) is smaller than desired. As a result, the consequent saccade has smaller amplitude, as the eyes do not reach the destination; catchup saccade is further programmed to compensate for the retinal slip error. The second possibility is that the original saccade command is normally programmed, the executed eye movement starts off with appropriate amplitude and velocity matrices, but it is interrupted by the intrusive signal that imposes the breaks in the eye position hence leading to a pause in the eye movement. The third possibility is that saccade in Whipple’s disease are slow in addition to being interrupted. To investigate these possibilities, we measured amplitudes and velocities of each segment of saccade (A1 and V1 in Figures 1D,F) as well as the desired amplitude and the maximum velocity (A and Vmax in Figures 1D,F). The prediction is that if saccades are programmed smaller, then their velocities (V1) are appropriately matched for the programmed amplitude (A1), hence the amplitude to velocity relationship, the main sequence, for A1 and V1 would fall within the normative range. However, the value of Vmax will be smaller for the overall amplitude (A), revealing abnormal main sequence for Vmax and A. In contrast, if saccades are normally programmed but prematurely interrupted then we expect normal amplitude to velocity relationship of the desired gaze shift and maximum velocity (Vmax and A), but velocity to amplitude relationship of staircase saccade (V1 and A1) will be above the normative range. Figure 2A depicts such comparison where the amplitudes of the saccade are plotted on the *x*-axis while velocities are plotted on the *y*-axis. The filled circles show Vmax to A relationship, while the open circles show V1 to A1 relationship of the segmented saccades. The amplitude to velocity relationship for segmented saccades fall along the lower margin of normative value, but the relationship of desired amplitude to velocity relationship falls below desired values. This phenomenon supports the first possibility for the pathomechanisms of abnormal horizontal saccades in Whipple’s disease that is saccades are mal-programmed, their amplitude is smaller than desired, and they are followed by series of “catch-up” saccades (staircase) to accomplish the desired gaze orientation. We found that the number of “catch-up” saccades per gaze shift ranged between 1 and 5; with a mean value of 2.7 ± 1.0.

### Disconjugacy Analysis

Each segment comprising the staircase horizontal saccade was disconjugate. The disconjugacy in the staircase saccades can be explained by two possible mechanisms. One, disconjugacy is due to an uneven central command to the right and left eye in the presence of strabismus. The second possibility is that disconjugacy in the amplitude is due to the uncertain timing of putatively intrusive signal that might have led to an early break in saccade trajectory. The first possibility also predicts a systematic relationship between the saccade amplitude and the amount of disconjugacy. We compared disconjugacy (the difference between the saccade amplitude of the right and left eye) with corresponding conjugate amplitude (mean amplitude of right and left eye). There was a positive correlation, with the slope of linear fit was 2.5 and intercept was 1.27. The correlation coefficient was 0.45 (Figure 2B). The results suggest the amount of disconjugacy increased with increasing saccade amplitude.

### Gaze-Evoked Nystagmus

**Figure 3:**
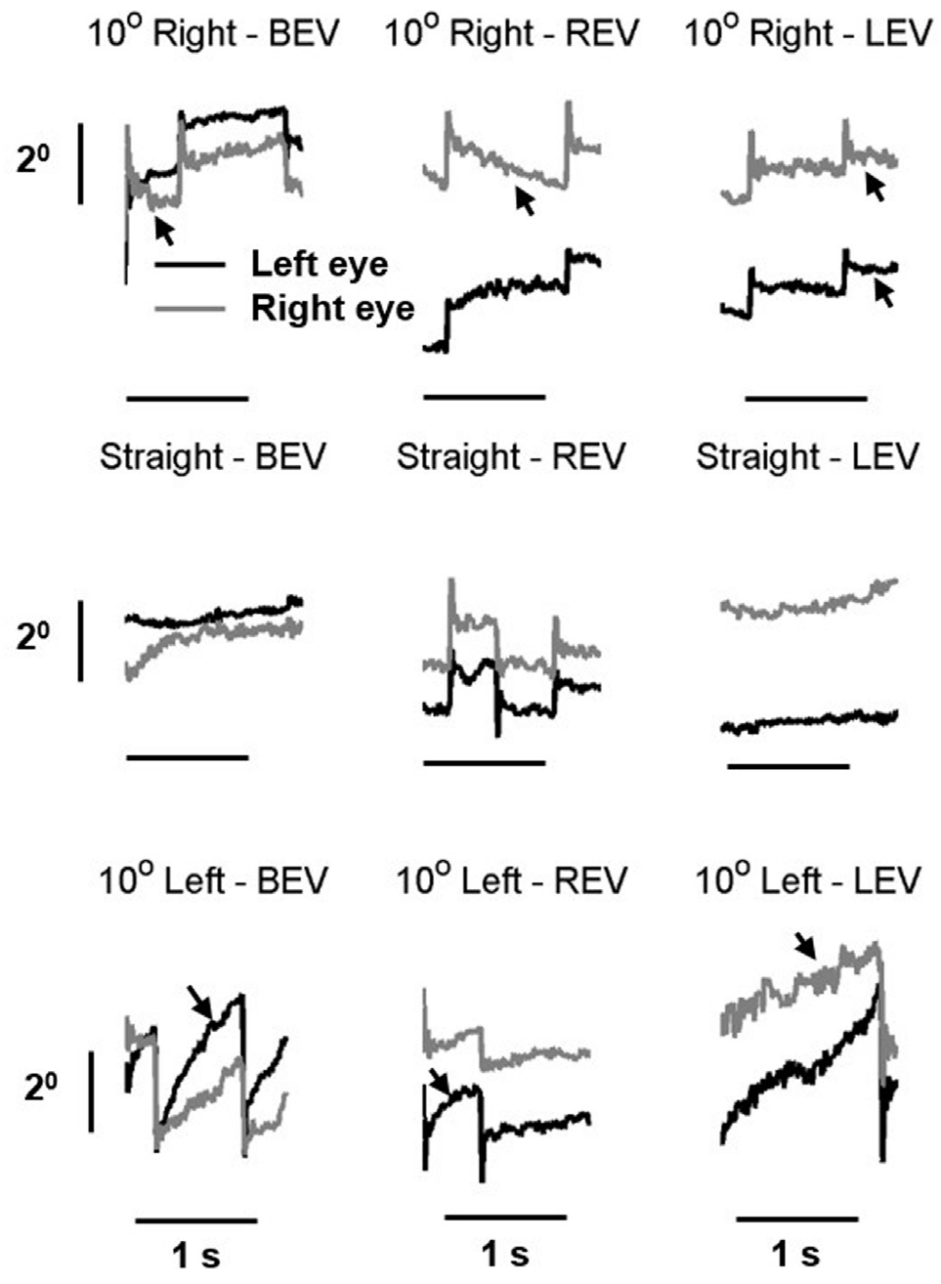
Example of gaze-evoked nystagmus in right eye viewing, left-eye viewing, and both eyes viewing condition. Gray line is right eye while black is the left eye. Eye positions are plotted on the *y*-axis and corresponding time is plotted on the *x*-axis. The arrows depict velocity-decreasing characteristics of the nystagmus waveforms.

Our patient also had gaze-evoked nystagmus. The slow phase of the nystagmus had velocity-decreasing waveform (arrows in Figure 3). The unique aspect was that the slow-phase eye velocity of the gaze-evoked nystagmus was disconjugate, more pronounced during monocular viewing condition (Figure 3). It is noteworthy that in addition to the drifts comprising the slow phase of the gaze-evoked nystagmus, there was a prominent post-saccadic drift of the covered eye during monocular viewing condition.

**Figure 4:**
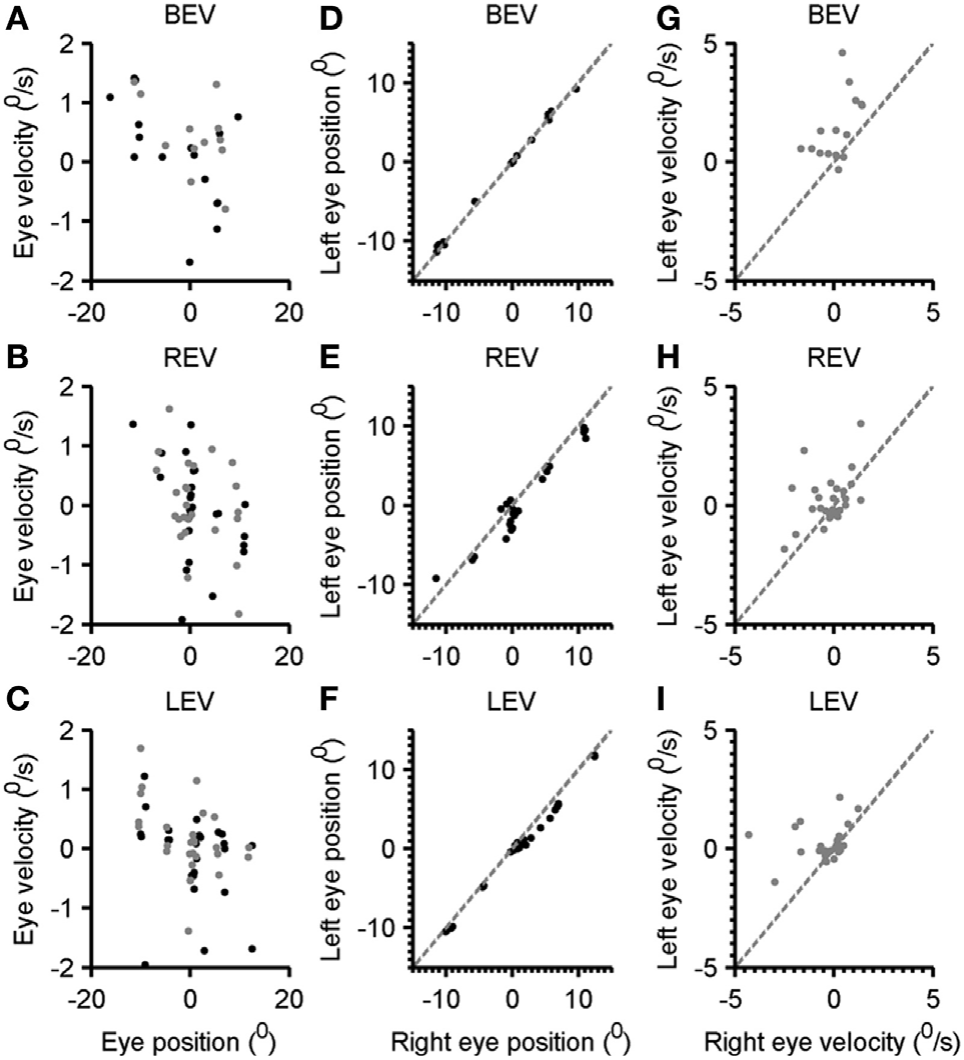
Comparison of the eye velocity with eye position in both eyes viewing, right eye viewing, and left-eye viewing condition **(A–C)**. Gray symbols are right eye while black symbols depict the left eye. Panels **(D–F)** depict the comparison of left-eye position, and right eye position gray dashed line is an equality line. Each black symbol depicts one eccentric position. Panels **(G–I)** illustrate a comparison of left-eye velocity (*y*-axis) with the right eye velocity (*x*-axis). Each symbol depicts one drift. Gray dashed line is an equality line.

The subsequent analysis depicts the quantitative summary of kinematic properties of gaze-evoked nystagmus at various eye-in-orbit positions (Figure 4A). Each data-point in Figure 4A depicts one drift, black symbol depicts right eye, and the gray symbol is the left eye. The trend is that with rightward eye positions (positive value on the *x*-axis) show leftward drift (negative eye velocity) and *vice versa* for the leftward gaze positions. Such relationship has a slope of 0.06 and correlation coefficient of 0.31 for the right eye and 0.09 (slope) and 0.24 (correlation coefficient) for the left eye. The comparable relationship of eye velocity and amplitude was seen in right and left eye viewing conditions (Figures 4B,C respectively). The slope of the fitted function for the left eye during right eye viewing condition is −0.1, and for the right eye, it was −0.08. The correlation coefficient for this relation was 0.33 for the right eye and 0.2 for the left eye. During left-eye viewing condition, the slope of this relationship was −0.04 for the right eye and −0.03 for the left eye. The correlation coefficient was 0.06 and 0.09 for right and left eyes, respectively.

To assess the level of disconjugacy and the dependence of eye position, we compared the right eye position versus the left-eye position at various gaze eccentricities. As illustrated in Figure 4D both eyes were well aligned as all data-points (each depicting the eye position at given eccentricity) fell on the gray dashed line (equality). The slope and intercept of such relationship were 0.9 and 0.1, while the correlation coefficient was 0.99. In contrast, during right eye viewing condition the right eye position data-points frequently fell below the equality line suggesting that the right eye typically overshoots further to the right, and it normally would be followed by leftward drift (Figure 4E). The slope of comparison of left and right eye position in right eye viewing condition was 0.9 and intercept was 0.7; the correlation coefficient was 0.95. During left-eye viewing condition the data-point also frequently fell below the equality line suggesting that the right eye shifted further to the right (Figure 4F). The slope of such relationship was 0.9 and intercept was 0.8, while correlation coefficient was 0.99. It is important to note that during rightward gaze positions the disparity was larger compared to all gaze conditions. In subsequent analysis, we compared the disconjugacy in drift velocity. As illustrated in Figure 4G, both eye velocities were robustly uneven suggesting disconjugacy in drift velocity despite consistent eye-in-orbit orientation during eccentric gaze positions in both eyes viewing condition. The slope and intercept of such relationship were 0.05 and 1.65, while the correlation coefficient was 0.03. The comparable finding was noted during right- and left-eye viewing conditions (Figures 4H,I). The slope of comparison of left and right eye velocities in right eye viewing condition was 0.4 and intercept was 0.34; the correlation coefficient was 0.2. The slope of the right and left-eye velocity relationship was 0.14 and intercept was 0.28, while correlation coefficient was 0.05.

## Discussion

The classic features of the central nervous system Whipple’s disease are pendular eye oscillations synchronized with the oscillatory jaw movements—called oculo-masticatory myorhythmia (16). Akinetic rigid forms of parkinsonism can be a manifestation of Whipple’s disease (8, 9, 17, 18). These features of the central nervous system Whipple’s disease can also present with slowing of vertical saccades, hence mimicking progressive supranuclear palsy (8, 9). In addition to up-gaze limitation, we found that patient with Whipple’s disease also had hypometric horizontal saccades comprised of multiple interruptions (staircase saccades), gaze-evoked nystagmus, and esotropia. These features supported a likelihood of abnormal cerebellar control. In subsequent sections, we will discuss the physiology of the phenomenology seen our patient with the central nervous system Whipple’s disease.

### Staircase Horizontal Saccades

There are two possible mechanisms for interruptions leading to staircase saccades. According to one phenomenology, the ongoing saccades are mal-programmed; each gaze shift is associated with a lower amplitude saccade making the gaze shift smaller compared to the desired position. Subsequently, a corrective saccade is generated, but it is also mal-programmed. The consequence of such deficits is the series of multiple small saccades leading to gaze shift to the desired location. This deficit suggests dysfunction of ocular motor vermis (19, 20). The second phenomenology also suggests a deficit in the saccadic system, but here the normally programmed saccades are interrupted by external intrusive signal leading to breaks in the ongoing saccade. Accordingly, the velocity of the segmented saccades would be higher compared to their corresponding amplitude. Instead, we found low normal saccade amplitude to velocity relationship of segmented horizontal saccades. Therefore, we propose that multiple interruptions of the horizontal saccades in a patient with Whipple’s disease could be due to mal-programmed saccades, favoring deficits in cerebellar control of eye movements.

We also found that each segmented saccade was associated with overshooting of one eye followed by a post-saccadic drift secondary due to esotropia as well as the pulse-step mismatch. This phenomenon led to disconjugacy in each segmented saccades followed by fusion. The amount of disconjugacy was proportional to the amplitude of the segmented saccade. Such systematic relationship between the amount of disconjugacy of the staircase and the staircase saccade amplitude is unlikely if the ongoing saccade was interrupted, but it is plausible for mal-programmed saccade. These results further supported our hypothesis that disconjugate and segmented horizontal saccades in our patient with Whipple’s disease were due to mal-programmed hypometric horizontal saccade, a characteristic of cerebellar lesion causing hypometria (21, 22).

### Gaze-Evoked Nystagmus

The gaze-evoked nystagmus is due to the insufficiency (leakiness) of the velocity-to-position neural integrator. Hence the direction of its slow-phase velocity reverses as the eye-in-orbit position shifts from one side of the null to the other, and the waveforms have velocity-decreasing characteristics (23, 24). Typical gaze-evoked nystagmus is seen in patients with focal or diffuse cerebellar deficit, leads to an impairment in the function of the neural integrator, and has conjugate slow phases (23). In contrast, our patient followed all characteristics of gaze-evoked nystagmus, but its slow phase was disconjugate. It is possible for the cerebellar disorder itself to cause disconjugate slow phase (19, 20, 25). However, our patient did not appear to have increased convergence tone esotropia reported in cerebellar disease patient, but had an esotropia due to horizontal gaze limitation during abduction. The difference in slow-phase velocity of the two eyes in our patient was more prominent during monocular viewing condition. We speculate that drifts of the esotropic eye that were most pronounced during monocular viewing condition interacted with the drifts that cause gaze-evoked nystagmus, possibly in the final common pathway for ocular motor control. Such interaction of post-saccadic drift and gaze-evoked nystagmus drift were more pronounced in the covered eye. Hence, we found a substantial disconjugacy in eye position and slow-phase velocity during monocular viewing condition.

In summary, unique presentation in our patient with Whipple’s disease further supports possible involvement of cerebellum, in addition to the brainstem and basal ganglia. This case further suggests that the central nervous system Whipple’s disease should not only be in the differential diagnosis of atypical forms of parkinsonism, such as progressive supranuclear palsy, but also multiple system atrophy. Finally, the analysis strategy proposed in this study can be used for differentiation of various disorders leading to staircase saccades, such as parkinsonian syndromes, cerebellar disorders, or deficits of brainstem saccade-generating circuits.

## Ethics Statement

The experiment protocol adhered declaration of Helsinki, and it was approved by the Cleveland Clinic institutional review board. The subject and his legal guardian gave written informed consent for the experiment and publication.

## Author Contributions

AS and FG: conceived idea, data collection, data analysis, writing, and editing manuscript.

## Conflict of Interest Statement

The authors declare that the research was conducted in the absence of any commercial or financial relationships that could be construed as a potential conflict of interest.

## Supplementary Material

Figure S1 Three panels depict 5-s long gaze-holding epochs in right, straight, and leftward gaze positions. The measurements are made during monocular viewing condition when the right eye (gray trace) was viewing. The red trace depicts (covered) left eye. Eye positions are plotted on the *y*-axis, while corresponding time is plotted on the *x*-axis.
