# Supplementary figures and images for: Novel Eye Movement Disorders in Whipple’s Disease—Staircase Horizontal Saccades, Gaze-Evoked Nystagmus, and Esotropia
Source: Front Neurol. 2017 Jul 11;8:321. doi: 10.3389/fneur.2017.00321 (PMC5504231; doi:10.3389/fneur.2017.00321)

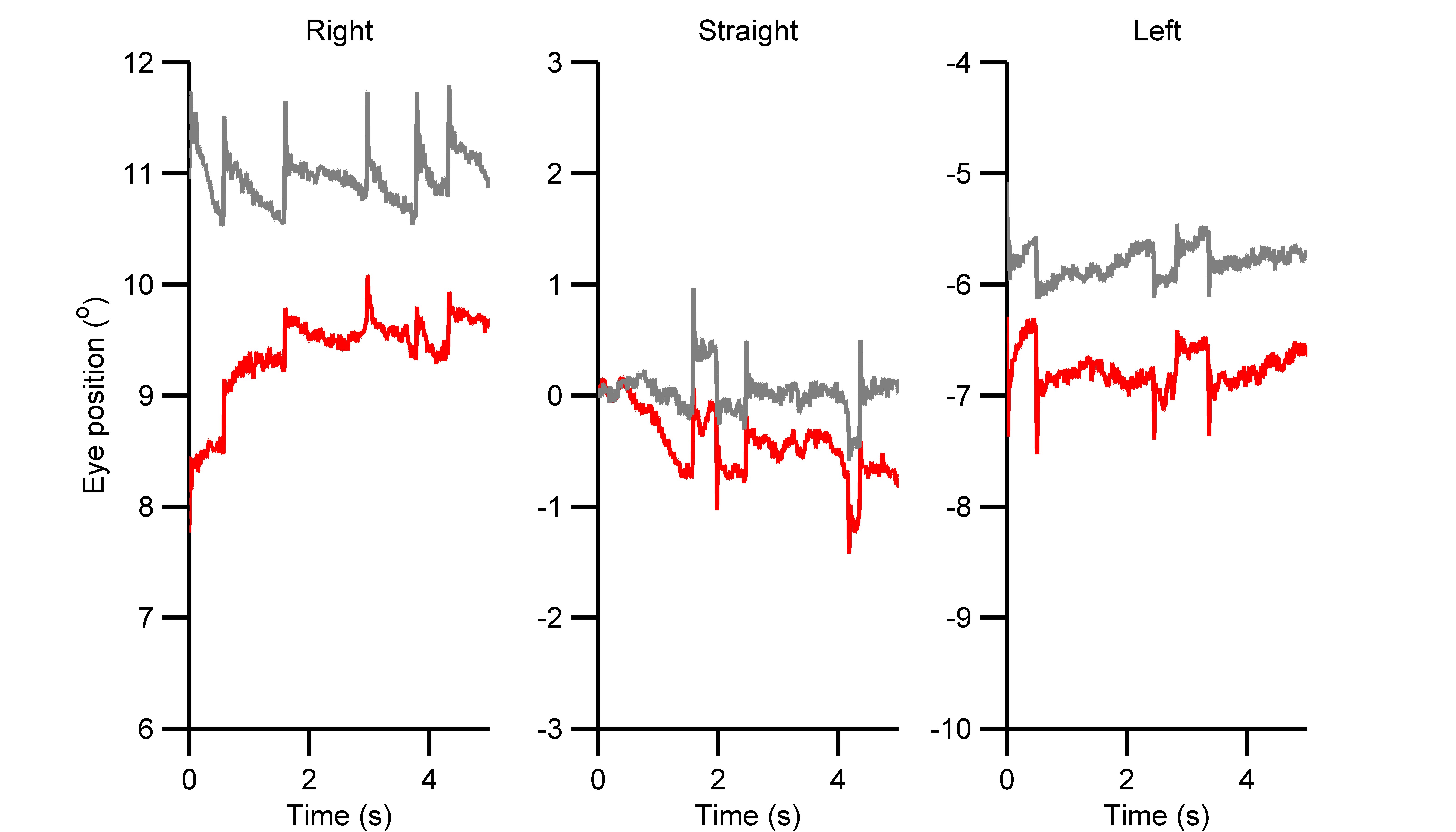

Supplement: Figure S1 — Three panels depict 5-s long gaze-holding epochs in right, straight, and leftward gaze positions. The measurements are made during monocular viewing condition when the right eye (gray trace) was viewing. The red trace depicts (covered) left eye. Eye positions are plotted on the y-axis, while corresponding time is plotted on the x-axis. [file Image_1.jpeg]
